# Medical Education in the Former Soviet Union: Opportunities in Armenia

**DOI:** 10.5334/aogh.2960

**Published:** 2020-08-13

**Authors:** Christopher Markosian, Shant Shekherdimian, Samuel S. Badalian, Lorky Libaridian, Ani Jilozian, Aline Baghdassarian

**Affiliations:** 1 Department of Neurological Surgery, Rutgers New Jersey Medical School, Newark, New Jersey, US; 2 Division of Pediatric Surgery, Department of Surgery, David Geffen School of Medicine at University of California, Los Angeles, Los Angeles, California, US; 3 Department of Obstetrics and Gynecology, State University of New York Upstate Medical University, Syracuse, New York, US; 4 Department of Internal Medicine, Harvard Medical School, Boston, Massachusetts, US; 5 Women’s Support Center, Yerevan, AM; 6 Departments of Emergency Medicine and Pediatrics, Virginia Commonwealth University School of Medicine, Richmond, Virginia, US

## Abstract

Medical education is a critical aspect of healthcare quality and thus requires sufficient investment to meet international standards. The Republic of Armenia, a nation that became independent of the Soviet Union in 1991, has not experienced significant advancement of its medical education system as the Western world has. In 2018, the country underwent a revolution to oust systematic corruption and transition to a true democracy, providing an opportunity for future efforts to improve medical education. The Armenian diaspora, which consists of approximately two to three times more individuals than the country’s population, includes healthcare professionals who are motivated and willing to contribute to the advancement of medical education. Assessing the perspectives of stakeholders is a key first step in this endeavor. We conducted a survey of recent medical graduates in Armenia, which revealed self-awareness of deficiencies in clinical, research, and leadership skills, desire to receive further training to improve these skills, and positive attitudes toward diaspora engagement. Thus, it is critical to incorporate a coordinated effort from the diaspora in addition to the local physician workforce, educational institutions, and government to bring about improvements in medical education in Armenia.

## Introduction

Medical education is one of the key determinants of healthcare quality. Underinvestment in the training of healthcare workers as well as incongruence of educational programs in the framework of healthcare systems and needs result in workforce shortages, especially in remote areas [1]. Though these tenets apply to all nations, countries formerly part of the Soviet Union have historically faced significant difficulty in solving these complex issues [2]. Despite the challenge, reforming medical education is one of the first steps toward optimization of a healthcare workforce’s service to a country’s population. We strongly believe that medical education reform should begin with seeking input from stakeholders, especially the learners, as we draw lessons from the Republic of Armenia.

Armenia, a landlocked country with a population of three million in the South Caucasus, has experienced extensive changes in its healthcare system since its independence from the Soviet Union in 1991 [3]. However, medical education has not evolved from its former strategies. As a post-Soviet nation, healthcare, and a majority of Armenia’s flagship higher education institutes, are state-run with administrations closely linked with acting political leaders. Systematic corruption throughout the government stagnated the progress of the country and remained an obstacle to not only healthcare, but also the evolution of medical education as experienced by the Western world. Only one of its seven institutions offering undergraduate medical education follows the Bologna process to comply with international standards [4]. Furthermore, there are currently no standards regulating residency educational curricula or residency positions available for different specialties. Knowledge gaps have even been described among some physicians in the country [5].

Geopolitical events in a country can pave the path toward a future with a positive outlook in many aspects, including healthcare. One such example is the 2014 political revolution in Ukraine, another former nation of the Soviet Union. This led to the replacement of the corrupt elite in decision-making roles and provided the state with an opportunity to modernize the country. Its Ministry of Health has begun implementing numerous healthcare reforms, including those related to medical education [6]. A similar scenario is currently unfolding in Armenia.

In 2018, Armenia underwent a populist anti-corruption campaign known as the Velvet Revolution. The dramatic and unexpected transition from an oligarchy placed Armenia on the international stage as a country with hope for true democracy, prompting it to be chosen as “Country of the Year” by *The Economist* [7]. Post-revolution Armenia serves as an opportunity for the country to modernize its medical education. The country’s large diaspora, consisting of about seven to nine million Armenians in approximately 81 countries, includes healthcare professionals who are willing and motivated to contribute and share their expertise [8,9]. During the current COVID-19 pandemic, diaspora physicians have been engaging with the Ministry of Health through the Office of the High Commissioner for Diaspora Affairs to offer assistance via an arguably more coordinated approach than in previous crises. This serves as a sign of institutional receptiveness to diaspora engagement and an effective approach by the diaspora in collaborating with stakeholders to improve healthcare in Armenia.

The first step in systematically improving medical education in Armenia is to receive input from physicians who have trained in and currently work in the country to strategically align efforts with perceived needs. A survey of recent medical graduates from three public medical centers in Yerevan, Armenia revealed that they possess insight regarding the shortcomings of the educational system as well as their own knowledge and skills (Table 1). Moreover, they are not only willing to dedicate extensive time to improving their skills, but also welcoming of capacity building from their compatriots abroad.

**Table 1:** Recent medical graduates’ overall characteristics and work description (Yerevan, Republic of Armenia, 2018).

Variable (*n* = 51)		Percent (%)^a^

Gender (*n* = 50)	Female	86.0
	Male	14.0
Age (*n* = 49)	22–25	16.3
	26–30	55.1
	31–35	20.4
	36–40	4.1
	41–45	4.1
Medical university (*n* = 47)	Yerevan State Medical University	89.4
	Haybusak University of Yerevan	8.5
	Mkhitar Gosh Armenian-Russian International University	2.1
Residency graduation year (*n* = 32)	2006–2011	3.1
	2011–2015	31.3
	2016–	65.6
Residency status (*n* = 49)	Completed	53.1
	In progress	46.9
Work location (*n* = 41)	Urban – Yerevan	78.0
	Urban – Other	19.5
	Rural	2.4
Clinical work setting (*n* = 40)	Hospital	70.0
	Outpatient clinic	25.0
Non-clinical activities (*n* = 40)	Medical education	22.5
	Administration	5.0
	Laboratory research	2.5

^a^ This value represents valid percent (i.e. missing answers were excluded from the calculation).

## Revealing Professional Needs

**Figure 1:**
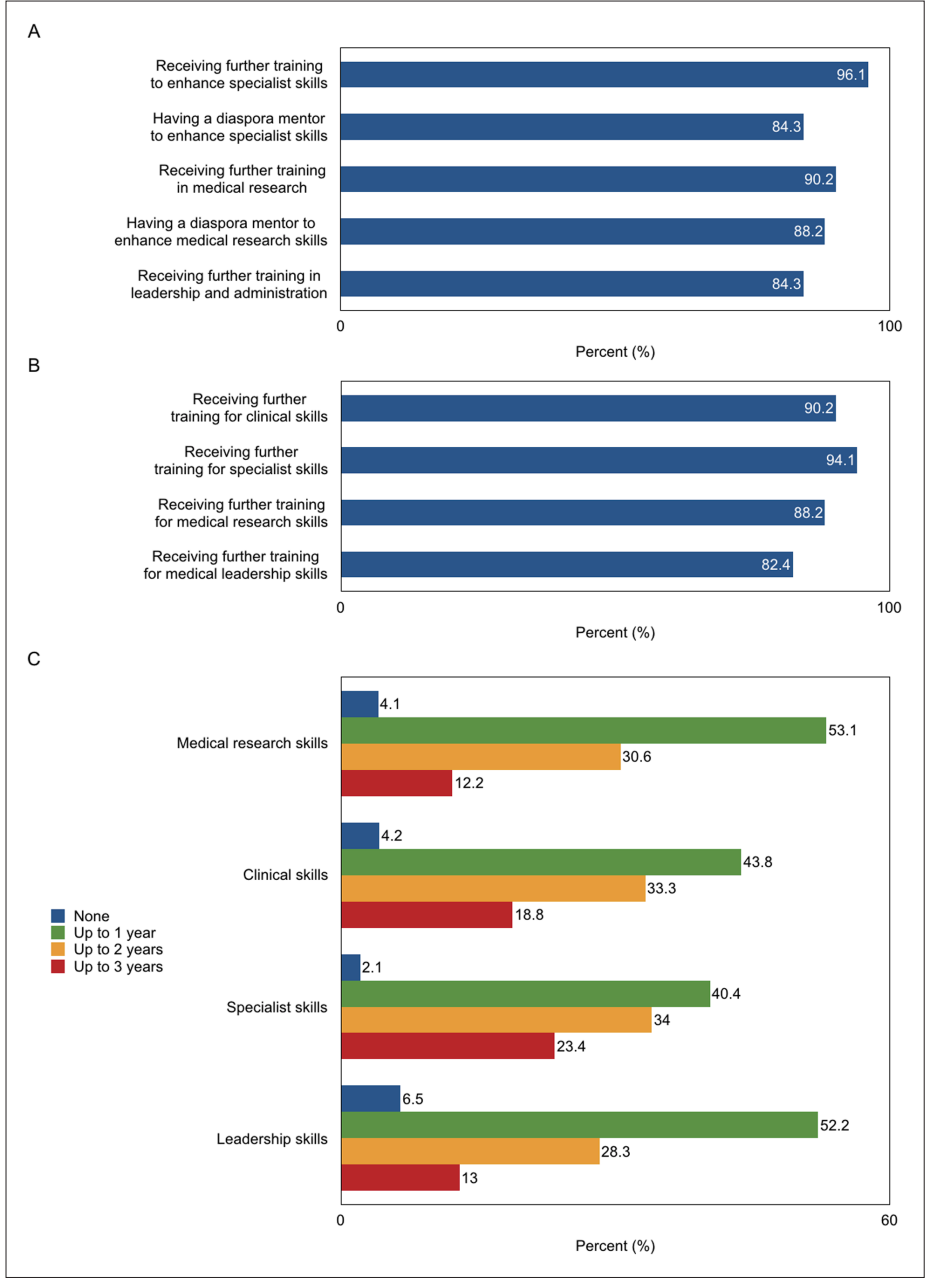
Recent medical graduates’ **(A)** perceived benefit of various medical activities, **(B)** interest in furthering their knowledge and skills, and **(C)** time willing to dedicate to training for various skills (Yerevan, Republic of Armenia, 2018). (Note: Reported percent represents actual percent i.e. missing answers were included in the calculation).

Higher education in Armenia, including medical education, continues to follow the rote learning methodology implemented during the Soviet Union. The curriculum lacks clinical application and independent practice opportunities necessary to develop critical thinking skills and clinical decision-making [10]. Awareness of these weaknesses and openness to addressing these limitations are evident, as almost all participants in our survey were interested in improving their broad and specialty-specific clinical skills (90.2% and 94.1%, respectively), and believed that they would benefit from additional training in their respective specialties (96.1%) (Figures 1A, 1B). Furthermore, learning how to plan, conduct, share, and comprehend research – which has become an essential component of Western medical education – is an overlooked component in the training of Armenian physicians. Almost all participants lacked previous training in medical research (95.8%), yet most believed that there is value in it (90.2%) (Figure 1B). A vast majority has voiced a willingness to spend up to 1–3 years in improving these skills (Figure 1C). Though these findings reinforce a grim reality, they point toward an optimistic future for the healthcare workforce if appropriate steps are taken to address them.

Interprofessional care has become an integral part of medicine in the 21^st^ century. This has prompted the Institute of Medicine to state that academic health centers “must be leaders and must develop leaders, at all levels” to improve health in innovative ways [11]. Consequently, medical schools have begun to implement leadership components in their educational curricula [12]. A majority of participants in our survey (84.3%) believed that they would benefit from further education and training to enhance their skills as leaders and administrators (Figure 1B). Most were interested in improving their leadership skills (82.4%) and were willing to devote 1–3 years to obtain medical leadership training (93.5%) (Figures 1A, 1C). This data, in combination with current trends in Western medical education, calls for the need to incorporate leadership curricula in local medical universities.

## Welcoming Diaspora Involvement

The incorporation of a diaspora to the development of a country has been demonstrated to be critical [13]. Diaspora knowledge networks are especially crucial in promoting the “brain gain” of a home country [14]. The vast majority of Armenian citizens believe that the diaspora is a positive force in the development of the country [8]. The Armenian diaspora has made numerous healthcare contributions to the country: Short-term mission trips to various villages, conferences between diaspora and local healthcare professionals, collaborations with local physicians on conducting research and organizing continuing medical education courses [15]. However, these efforts tend to focus on short-term interventions, they are largely uncoordinated, and their impact is rarely measured. International health collaborations can be optimized through an understanding of the perspectives of stakeholders. Most participants in our survey (81.8%) believed that the diaspora has a role in the healthcare system of Armenia: Such as providing funds and equipment, contributing knowledge and expertise in providing patient care, improving medical education and training, and/or contributing to the healthcare system (Table 2). Mentorship from diaspora physicians is also valued in augmenting various skills at the level of the individual (Figure 1B). While many local physicians expect and welcome help from the diaspora, it is essential for this assistance to fit in the strategic plan through a well-coordinated, transparent effort.

**Table 2:** Recent medical graduates’ perspectives on diaspora roles in Armenia’s healthcare system (Yerevan, Republic of Armenia, 2018).

Variable (*n* = 51)	Strongly agree (%)	Agree (%)	Neutral (%)	Disagree (%)	Strongly disagree (%)

Provide funds and equipment (*n* = 46)	50.0	34.8	6.5	6.5	2.2
Contribute knowledge and expertise in providing patient care (*n* = 46)	65.2	23.9	6.5	0.0	4.3
Help improve medical education and training (*n* = 47)	61.7	31.9	4.3	2.1	0.0
Contribute to healthcare system (*n* = 43)	53.5	30.2	16.3	0.0	0.0
None (*n* = 33)	3.0	0.0	15.2	30.3	51.5

^a^ This value represents valid percent (i.e. missing answers were excluded from the calculation).

## Conclusions

With a better understanding of the perceptions of recent medical graduates, future interventions can be proposed to address deficiencies in medical education. The younger generation of physicians in Armenia is aware of its weaknesses, is willing to put in the work for improvement, and is receptive to diaspora contributions in capacity building. These physicians are in need of educational guidance and mentorship, providing a key role for Armenians abroad. Thus, it is critical to incorporate a coordinated effort from the diaspora in addition to the local physician workforce, educational institutions, and government to precipitate improvement in this nation of newfound opportunities.
